# Docetaxel: an alternative taxane in ovarian cancer

**DOI:** 10.1038/sj.bjc.6601495

**Published:** 2003-12-17

**Authors:** N Katsumata

**Affiliations:** 1 Department of Medical Oncology, National Cancer Centre Hospital, 5-1-1 Tsukiji, Chuo-ku, Tokyo 104-0045, Japan

**Keywords:** docetaxel, taxanes, ovarian cancer, chemotherapy

## Abstract

The taxanes paclitaxel and docetaxel are potent chemotherapeutic agents that block tubulin depolymerisation, leading to the inhibition of microtubule dynamics and cell cycle arrest. Although docetaxel and paclitaxel share a mutual tubulin binding site, mechanistic and pharmacological differences exist between these agents. For example, docetaxel has increased potency and an improved therapeutic index compared with paclitaxel, and its short 1-h infusion offers a substantial clinical advantage over the prolonged infusion durations required with paclitaxel. In clinical studies, docetaxel monotherapy demonstrated good response rates and an acceptable toxicity profile in both paclitaxel- and platinum-refractory ovarian cancer patients. In particular, neurotoxicity — a dominant side effect with both paclitaxel and cisplatin — occurs at a low incidence with docetaxel, making docetaxel a promising agent for combining cisplatin and other platinum compounds. In Phase II studies, the combination of docetaxel with either cisplatin or carboplatin has yielded impressive response rates of 69–74 and 81–87%, respectively. Furthermore, Phase III data suggest that docetaxel–carboplatin and paclitaxel–carboplatin are similarly efficacious with respect to progression-free survival and clinical response, although neurotoxicity occurs more frequently with the paclitaxel regimen. While paclitaxel–carboplatin remains the standard treatment for the management of advanced ovarian cancer, docetaxel–carboplatin appears to be a promising alternative, particularly in terms of minimising the incidence and severity of peripheral neuropathy.

Ovarian cancer accounts for nearly 4% of cancers among women and is the leading cause of gynaecological cancer death in the USA (American Cancer Society, 2003). Indeed, the American Cancer Society estimates that during 2003 a total of 25 400 new cases of ovarian cancer will be diagnosed in the USA, and that almost 14 300 US women will die from this disease (American Cancer Society, 2003). Platinum-based chemotherapy has been the cornerstone of therapy for advanced ovarian carcinomas since the activity — in the early 1980s — of cisplatin-based regimens in ovarian cancer was first reported (Decker *et al*, 1982; Lambert and Berry, 1985; Kaye, 2000). Subsequently, platinum-based combination therapies have been shown to achieve higher clinical response rates and longer progression-free intervals than alkylating agents alone, or nonplatinum regimens, although the evidence for overall survival benefit with such regimens — in cases of advanced ovarian cancer — is less compelling (Aabo *et al*, 1998). More recently, two large randomised trials, one conducted by the Gynecologic Oncology Group (GOG) and the other by the European Organisation for Research and Treatment of Cancer (EORTC), have shown that administration of the taxane paclitaxel in combination with cisplatin significantly improves the duration of progression-free survival and overall survival in women with advanced epithelial ovarian cancer compared with cisplatin–cyclophosphamide therapy (McGuire *et al*, 1996; Piccart *et al*, 2000). Paclitaxel–platinum combinations are therefore replacing platinum-alkylating agent regimens as standard first-line therapy in advanced ovarian cancer (Kaye, 2000). However, since both paclitaxel and cisplatin are neurotoxic, such combinations are associated with a high degree of neuropathy. Two recently published large randomised trials have shown that paclitaxel–carboplatin achieved comparable efficacy and less toxicity compared with paclitaxel–cisplatin (du Bois *et al*, 2003; Ozols *et al*, 2003). It would therefore appear that paclitaxel–carboplatin may provide another first-line chemotherapy regimen for the treatment of advanced ovarian cancer.

Docetaxel is a newer member of the taxoid family, derived by a semisynthetic process from the needles of the European Yew tree *Taxus baccata* (Denis *et al*, 1990). This agent has shown significant activity in a variety of cancers including breast, lung, ovarian, head and neck, and gastric cancers. Like paclitaxel, docetaxel acts as a spindle poison, promoting microtubulin assembly and stabilising the polymers against depolymerisation, leading to the inhibition of microtubule dynamics and cell cycle arrest (Ringel and Horwitz, 1991). Although docetaxel and paclitaxel share a mutual tubulin binding site, mechanistic and pharmacological differences exist. For example, preclinical studies have shown that — compared with paclitaxel — docetaxel is a stronger promoter of tubulin polymerisation *in vitro*, has a longer intracellular half-life and demonstrates greater activity in some tumour models (Barasoain *et al*, 1991; Ringel and Horwitz, 1991; Bissery *et al*, 1995).

Docetaxel has demonstrated potent *in vitro* and *in vivo* cytotoxic activity against a range of tumour types, particularly ovarian cancer. Indeed, docetaxel was found to be 1.2–2.6 times more cytotoxic than paclitaxel and over 1000 times more cytotoxic than cisplatin or etoposide in ovarian carcinoma cell lines (Kelland and Abel, 1992; Engblom *et al*, 1997). Docetaxel has also been shown to act synergistically with cisplatin and carboplatin in epithelial ovarian cancer *in vitro*, and to have potent cytotoxic activity in ovarian cell lines that are resistant to these agents (Kelland and Abel, 1992). Furthermore, there is incomplete cross-resistance between paclitaxel and docetaxel in a range of *in vitro* human tumour cell lines (including ovarian) (Hanauske *et al*, 1992); and in clinical trials, docetaxel 75 or 100 mg m^−2^ every 3 weeks has been found to be an active second-line agent in patients refractory to paclitaxel-based regimens (Verschraegen *et al*, 2000).

Docetaxel and paclitaxel also have substantially different toxicity profiles. Of particular note, docetaxel is associated with only minimal neurotoxicity, which has prompted interest in the use of this agent as an alternative to paclitaxel for inclusion in platinum-based regimens for the management of advanced ovarian cancer (Markman *et al*, 2001; Vasey on behalf of the Scottish Gynaecological Cancer Trials Group, 2002). In the light of these observations, this paper examines clinical experience to date with docetaxel and discusses the potential of this drug as an alternative to paclitaxel in the management of ovarian cancer.

## DOCETAXEL MONOTHERAPY

### Phase I trials

The clinical efficacy of docetaxel was first reported in Phase I studies in patients with a range of solid tumours (including ovarian cancer) resistant to standard chemotherapy in use at the time of these early trials (Cortes and Pazdur, 1995). These studies identified a short 1-h infusion as the optimal means of delivering docetaxel (Aapro *et al*, 1992; Bissett *et al*, 1993; Extra *et al*, 1993; Cortes and Pazdur, 1995) — offering a substantial clinical advantage over paclitaxel, which requires longer infusion times (3 or 24 h). Neutropenia was the major toxicity reported with docetaxel in Phase I trials; this was dose- but not schedule-dependent (Cortes and Pazdur, 1995). Other side effects included mucositis, hypersensitivity reactions, asthenia and fluid retention, although fluid retention is now routinely prevented by the prophylactic administration of steroids (Cortes and Pazdur, 1995; Kaye *et al*, 1997; Piccart *et al*, 1997).

### Phase II trials

The safety and efficacy of docetaxel 100 mg m^−2^ administered every 3 weeks as a 1-h intravenous infusion have been evaluated in four Phase II trials in women with platinum-refractory advanced ovarian cancer. Two of these studies were multicentre European trials conducted by the Early Clinical Trials Group (ECTG) and the Clinical Screening Group (CSG) of the EORTC, and two were single-centre trials conducted in the USA by the MD Anderson Cancer Center (MDACC) and the Memorial Sloan–Kettering Cancer Center (MSKCC) (Aapro *et al*, 1994; Francis *et al*, 1994; Piccart *et al*, 1995; Kavanagh *et al*, 1996). A total of 340 patients were included, all of whom had been previously treated with cisplatin or carboplatin and had recurrent or progressive disease. A summary of the characteristics of the patients enrolled in these trials and their response to docetaxel therapy are provided in Table 1

**Table 1:** Efficacy of docetaxel 100 mg m^−2^ every 3 weeks in women with recurrent or progressive ovarian cancer previously treated with platinum compounds: results from four Phase II studies (adapted from Kaye *et al*, 1997)

	**Study**			
	**ECTG**	**CSG**	**MDACC**	**MSKCC**
*Patient characteristics*				
No. of patients				
Treated	132	124	59	25
Evaluable for efficacy	116	121	55	23
Median age [range] (years)	54 [30–75]	57 [35–76]	58 [26–70]	59 [36–73]
Interval since prior platinum therapy (% of patients)				
0–4 months	30	38	100	83
4–12 months	35	62	—	17
>12 months	35	—	—	—
				
*Response to therapy (evaluable population)*				
Response rates (% of patients)				
Complete response	3	7	5	0
Partial response	25	19	35	35
No change	41	36	38	43
Median response duration [range] (months)	6.7 [4.1–17.4]	5.8 [1.4–13.5]	4.5 [1–12]	5.0 [3–9]
Median survival (months)	8.4	10.4	10	8

CSG=Clinical Screening Group; ECTG=Early Clinical Trials Group; MDACC=MD Anderson Cancer Center; MSKCC=Memorial Sloan–Kettering Cancer Center.

.

Overall response rates across the four individual trials ranged from 26 to 40% (Kaye *et al*, 1997). When response data from the four trials were pooled, there were 14 complete responses and 79 partial responses among the 315 evaluable patients, giving an overall response rate of 30% (95% confidence intervals (CI): 19–36%) (Kaye *et al*, 1997). Importantly, docetaxel maintained this high response rate even in the most platinum-refractory patients, with an overall response rate of 28% (95% CI: 19–36%) in the 155 patients with a treatment-free interval of less than 4 months. The median duration of response and the median survival in the four individual trials ranged from 4.5 to 6.7 months and from 8 to 10.4 months, respectively. The overall response rates obtained with docetaxel in these four Phase II studies compare favourably with the 22% response rate reported with paclitaxel in a large population-based study in women with platinum-refractory disease (Trimble *et al*, 1993).

The toxicity profile of docetaxel was similar across the four trials and reflected observations made in the Phase I studies. Neutropenia was the most frequently reported grade III–IV toxicity (90–96% of patients) and was followed by severe fluid retention, which was experienced by 8–12% of patients. However, none of these studies included steroid prophylaxis, which has since been shown to reduce significantly the incidence and severity of fluid retention, and also the frequency of treatment discontinuation due to this adverse event. Consequently, routine premedication with a steroid (e.g. dexamethasone) has been incorporated in subsequent docetaxel studies. Other grade III–IV toxicities reported in the four Phase II trials in advanced ovarian cancer included acute hypersensitivity (7–10% of patients), diarrhoea (6–10%), dermatitis (4–8%) and stomatitis (0–5%). From these Phase II studies, it can be concluded that docetaxel demonstrates significant clinical activity against advanced ovarian cancer and has a different spectrum of toxicity to paclitaxel, which is commonly associated with neuropathy and myalgia.

### Phase II trials using low-dose docetaxel

As an alternative to administering prophylactic steroids to reduce the degree of fluid retention, Japanese studies have tended to use lower doses of docetaxel than those used in European and American trials. In a Phase I study conducted in Japan in patients with solid tumours, the maximum tolerated dose of docetaxel without premedication ranged between 70 and 90 mg m^−2^ (Taguchi *et al*, 1994). On this basis, the Japanese Phase II programme for docetaxel was initiated at a dose of 60 mg m^−2^. However, while this dose generated good response rates in women with breast cancer, results in ovarian cancer were disappointing (only one partial response and no complete responses in 36 evaluable patients) (Noda *et al*, 1994). In a subsequent Phase II pilot study, the dose of docetaxel was increased to 70 mg m^−2^ every 3 weeks in Japanese women with platinum-pretreated advanced ovarian cancer. This resulted in an acceptable tolerability profile and delivered a response rate of 24% in the 25 evaluable patients (Fujiwara *et al*, 1999).

The clinical efficacy and tolerability of docetaxel 70 mg m^−2^ every 3 weeks in advanced ovarian cancer have since been confirmed in a larger Phase II study in Japan (Katsumata *et al*, 2000). Here, 60 women previously treated with platinum-based therapies received a median of four courses of docetaxel, 98% of which were given without the need for dose reduction. Response was achieved in 25% of platinum-refractory patients (within 0–6 months of the platinum-free interval) and 33% of platinum-sensitive patients (within 6 and more months of the platinum-free interval); the overall response rate was 28% for all patients combined. Haematological effects were the main toxicities associated with therapy and were recorded at frequencies similar to those observed in European and US Phase II programmes. However, nonhaematological toxicities tended to be milder than had been reported with higher docetaxel dosages. In particular, there was a low incidence of severe hypersensitivity reactions or fluid retention, despite the fact that steroid prophylaxis was not given in this or any other Japanese Phase II trial. Given that the response rates achieved in this trial were similar to those achieved in the higher-dose European and US trials, reducing the docetaxel dosage to 70 mg m^−2^ may be the preferred chemotherapeutic approach in patients for whom steroid premedication is inappropriate.

## DOCETAXEL–PLATINUM: AN ALTERNATIVE FIRST-LINE THERAPY

### Overview of docetaxel–cisplatin trials

As mentioned previously, the superiority of paclitaxel–cisplatin regimens as first-line chemotherapy over cisplatin–cyclophosphamide therapy (the previous standard of care) has been established in two large randomised trials in women with advanced epithelial ovarian cancer (McGuire *et al*, 1996; Piccart *et al*, 2000). One of the major limitations of this combination is that both paclitaxel and cisplatin are neurotoxic, and co-administration of these two agents can result in a high incidence of peripheral neuropathy. This has led several groups, including the French Group d'Investigateurs Nationaux pour l'Etude des Cancers Ovariens (GINECO), the Russian RAMS group and the Scottish Gynaecological Clinical Trials Group (SGCTG), to evaluate the potential of docetaxel as an alternative taxoid to paclitaxel for use in combination with cisplatin in this patient population (Guastalla *et al*, 1999; Vasey *et al*, 1999; Gorbounova *et al*, 2000). In each of these studies, docetaxel 75 mg m^−2^ and cisplatin 75 mg m^−2^ were administered every 3 weeks for six courses with routine steroid premedication.

In an interim analysis of the Russian RAMS study, the overall rate of clinical response to docetaxel–cisplatin among the 38 evaluable patients was 73.6%, of which 42.1% were complete responses and 31.5% partial responses (Gorbounova *et al*, 2000); four patients experienced a pathological complete response. In the GINECO trial, docetaxel–cisplatin was associated with a pathological complete response in 21% of the 43 evaluable patients, and a disease-free survival of 16 months after a median 16 months follow-up (Guastalla *et al*, 1999). In both trials, docetaxel–cisplatin had an acceptable tolerability profile. No unexpected toxicities were reported (neutropenia was the most common adverse event) and the rates of neurological toxicity and fluid retention were low.

The SGCTG trial differed from the RAMS and GINECO studies in that patients were divided into two treatment cohorts: one receiving cisplatin 75 mg m^−2^ plus docetaxel 75 mg m^−2^ (*n*=49), the other receiving cisplatin 75 mg m^−2^ plus docetaxel 85 mg m^−2^ (*n*=51) (Vasey *et al*, 1999). In addition, the study was designed primarily to assess the toxicity of the docetaxel–cisplatin combination, its primary end point being the proportion of patients who discontinued therapy because of fluid retention. Only two-thirds of patients completed the full six courses of therapy, with half of all patient withdrawals being attributed to treatment-related toxicity. However, no patients withdrew because of fluid retention and only 14 patients (14%) developed peripheral oedema requiring diuretics, which confirmed previous reports that premedication with a 5-day course of corticosteroids reduces the severity of this adverse event. The incidence of moderate to severe peripheral neuropathy was low (6% grade III). Among the 39 patients who were available for assessment of clinical response after three or six cycles of chemotherapy, 38% had a complete response and 31% a partial response.

### Overview of docetaxel–carboplatin trials

There is now a large body of evidence to suggest that in patients with ovarian cancer, carboplatin provides comparable antitumour activity to cisplatin, but with significantly less toxicity when given as monotherapy or in combination with other agents (Aabo *et al*, 1998). The addition of carboplatin to a taxane regimen was expected to result in less emesis and neurotoxicity than cisplatin–taxane therapy, although concerns were expressed that the combined myelotoxicity of carboplatin and a taxane might result in significant myelosuppression, necessitating dose reduction. However, experience with paclitaxel–carboplatin has shown that the two agents can be given safely without reduction in the dosage of either component (Kaye, 2000; du Bois *et al*, 2003). Indeed, it appears that carboplatin-associated thrombocytopenia is reduced by co-administration of paclitaxel — an effect thought to occur at the level of the megakaryocyte rather than by a general pharmacokinetic interaction (Kaye, 2000). Given these promising results, a series of Phase I/II trials have been conducted to assess docetaxel–carboplatin regimens in this setting, and Phase III trials are underway.

### Phase I/II experience

In a recent Phase I trial of docetaxel and carboplatin as first-line therapy, 22 patients with ovarian cancer were given docetaxel as a 1-h infusion immediately followed by a 1-h infusion of carboplatin (Hatae *et al*, 2002). Dose-limiting toxicities of febrile neutropenia and grade IV diarrhoea were seen at the dose level of docetaxel 75 mg m^−2^ and carboplatin AUC 6. Pharmacokinetic data for docetaxel were similar to those reported for docetaxel administered as a single agent, and no pharmacokinetic drug–drug interactions were seen. The recommended doses were determined as docetaxel 75 mg m^−2^ plus carboplatin AUC 5 or docetaxel 70 mg m^−2^ plus carboplatin AUC 6.

**Figure 1:**
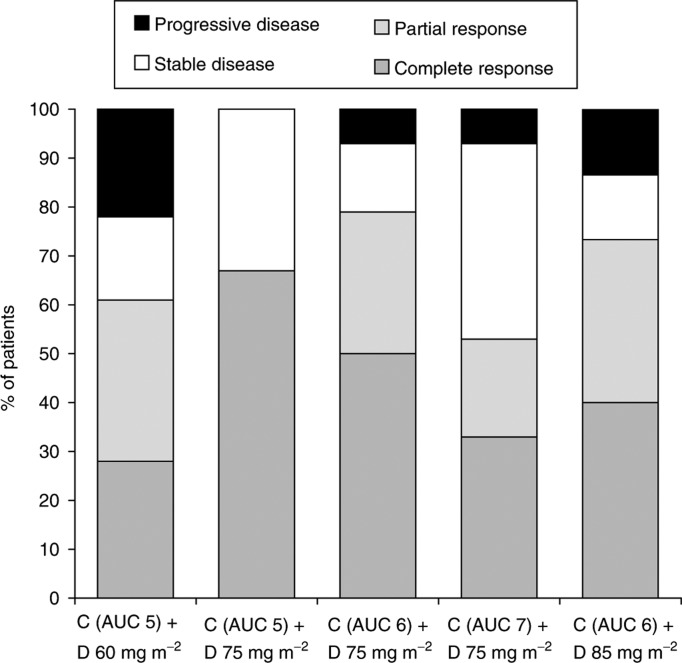
Response rates to first-line docetaxel (D)–carboplatin (C) therapy in a dose-finding study of D 60–85 mg m^−2^ and C AUC 5–7 in women with ovarian cancer (73 evaluable for response) (Vasey *et al*, 2001).

The efficacy and safety of docetaxel–carboplatin regimens as first-line therapy for epithelial ovarian cancer were first reported by the SGCTG group (Vasey *et al*, 2001). Their feasibility study included 139 eligible patients (median age 56 years; 79% FIGO stage III/IV at presentation) treated at one of five docetaxel–cisplatin dosage levels, with docetaxel doses ranging between 60 and 85 mg m^−2^, and carboplatin doses ranging between an area under the concentration–time curve (AUC) of 5 and 7 mg ml^−1^. Treatment was administered every 3 weeks for six planned cycles, with a 3-day prophylactic dexamethasone regimen. The overall clinical/radiological response rate was 66, and 75% of patients had a CA125 response. Median progression-free survival was 16.6 months at a median follow-up of 19 months. Response to therapy at each of the five dosage levels is shown in Figure 1

**Figure 2:**
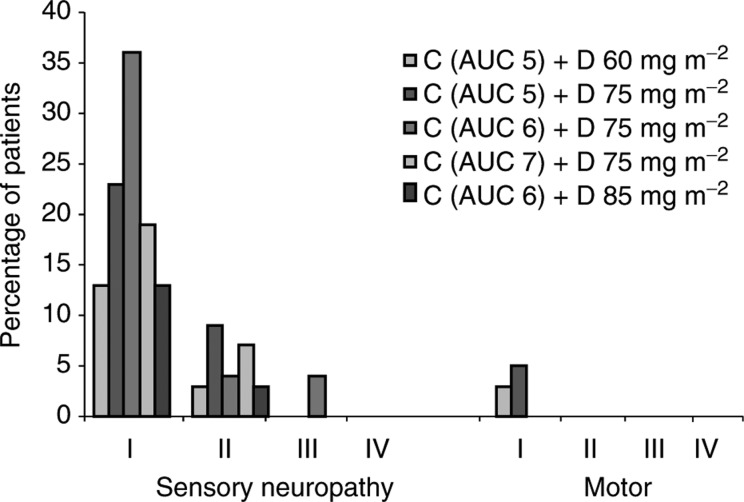
Incidence of neuropathic toxicities reported during first-line docetaxel (D)–carboplatin (C) therapy in a dose-finding study of D 60–85 mg m^−2^ and C AUC 5–7 in women with ovarian cancer (139 evaluable for safety) (Vasey *et al*, 2001).

. The incidence of neurotoxicity was extremely low and no patients were removed from the study as a direct result of this side effect. Indeed, grade II/III sensory neurotoxicity was reported by fewer than 6% of patients and there were no cases of motor neuropathy of severity greater than grade I; these rates of neuropathy are substantially lower than those reported with paclitaxel–carboplatin regimens. A summary of the neuropathic toxicities reported at the various dosage levels is provided in Figure 2

. As anticipated, neutropenia was the major dose-limiting toxicity. CTC grade IV neutropenia occurred in 75% of patients; however, in only 4% of patients was this effect associated with sepsis, and prophylactic antibiotics or growth factors were not routinely required. Grade IV thrombocytopenia was seen in only 4.2% of patients and there were no cases of thrombocytopenic haemorrhage, which suggests that the platelet-sparing effect of paclitaxel when given with carboplatin also extends to docetaxel and is therefore most probably a class effect of the taxoids. On the basis of these results, the dosage regimens recommended by the SGCTG for further trials were docetaxel 75 mg m^−2^ plus carboplatin AUC 5 or 6.

The activity and safety of docetaxel 70–75 mg m^−2^ plus carboplatin to AUC 5–6 every 3 weeks in women with stage III–IV ovarian cancer have been confirmed in three other Phase II studies involving a total of 66 women, 50 of whom were chemonaïve and 16 of whom had received prior platinum-based therapy (Table 2

**Table 2:** Efficacy and safety of docetaxel (D) 60–75 mg m^−2^ plus carboplatin (C) AUC 5–6 every 3 weeks in women with recurrent or progressive ovarian cancer (OC): results from four Phase II studies (Meyer *et al*, 1999; Kolevska *et al*, 2001; Markman *et al*, 2001; Vorobiof *et al*, 2001)

		**No. of patients evaluable for**		**Patient characteristics at presentation**	**Response rate (% of evaluable patients)**			**Length of follow-up, median [range]**	**Progression-free survival, median [range]**	**Toxicity (no. of patients)**
**Reference**	**Regimen**	**Safety**	**Efficacy**		**CR**	**PR**	**Overall**			
Markman *et al* (2001)	D 60 mg m^−2^+C AUC 6 every 21 days for 6 cycles	50	42	Chemotherapy-naïve (94%) and platinum-pretreated (6%) patients with OC (12% stage I–II; 88% stage III–IV)	NS	NS	81	21+ months [12+ to 41+]	>16 months	Grade IV neutropenia (32); neutropenic fever (8); grade III thrombocytopenia (2); hypersensitivity (17); peripheral neuropathy (3)
Kolevska *et al* (2001)	D 70 mg m^−2^+C AUC 6 every 21 days for 6 cycles	19	15	Chemotherapy-naïve patients with suboptimally debulked stage IIIc (63%) or IV (37%) OC	27	53	87^a^	8.9 months [0.6–26.4]	13.1 months	Grade III/IV toxicities:^b^ neutropenia (15); febrile neutropenia (2); nausea (2); vomiting (1); anaemia (2); thrombocytopenia (3) oedema (1); weight loss (1); dehydration (1); DVT (3); diarrhoea (2)
Meyer *et al* (1999)	D 75 mg m^−2^+C AUC 5every 21 days for 6 cycles	27	27	Chemotherapy-naïve (41%) and platinum-pretreated (59%) patients with stage III–IV OC	52	29	81	NS	NS	Neutropenia (grade II/III, 24); thrombocytopenia (grade II/III, 7); neuropathy (grade II, 2)
Vorobiof *et al* (2001)	D 75 mg m^−2^+C AUC 6every 21 days for 6 cycles	20	11^c^	Chemotherapy-naïve patients with stage III–IV OC	46	36	82	NS	NS	Grade III–IV toxicities included anaemia, leucopenia, neutropenia, thrombocytopenia, nausea and vomiting

a Includes one minor response.

b Data presented as number of episodes.

c Includes patients with measurable disease and excludes those only evaluable for CA125 response.

) (Meyer *et al*, 1999; Kolevska *et al*, 2001; Vorobiof *et al*, 2001). In these studies, 27–52% of patients achieved a complete response and 29–53% a partial response following docetaxel–carboplatin therapy, with overall response rates ranging from 81 to 87% (Table 2) (Meyer *et al*, 1999; Kolevska *et al*, 2001; Vorobiof *et al*, 2001). These response rates suggest that this docetaxel–carboplatin regimen is at least as effective as docetaxel–cisplatin regimens.

In all of the studies, neutropenia was the major toxicity. Neurotoxicity was reported in two of the three studies, but the incidence was very low: Kolevska *et al* (2001) reported grade I neuropathy in seven out of 19 patients, whereas Meyer *et al* (1999) reported grade II neuropathy in two out of 26 patients and grade I neuropathy in 15 out of 26 patients (no cases of grade III or above). Survival and quality of life data have been reported for one of the three studies — Kolevska and colleagues found that first-line therapy with docetaxel 70 mg m^−2^ plus carboplatin to AUC 6 every 21 days was associated with a median progression-free survival of 13.1 months in women with cancer of the ovaries, fallopian tube or peritoneum (at the time of the report, median overall survival had not been reached: 9.2+ months) (Kolevska *et al*, 2001). Over the course of the study, 50% of patients experienced a 10-point improvement in the Functional Living Index: Cancer (FLIC) quality of life questionnaire, with 25% experiencing no change and 25% experiencing a 10-point deterioration in FLIC score (Kolevska *et al*, 2001).

Markman *et al* (2001) have reported similarly high response rates in a Phase II study employing a lower 60 mg m^−2^ dose of docetaxel (Table 2). A total of 50 women with cancer of the ovary and fallopian tube and primary cancer of the peritoneum were treated with docetaxel 60 mg m^−2^ plus carboplatin AUC 6 every 3 weeks for six cycles. The vast majority of patients were chemonaïve (94%) and had stage III–IV disease (88%). Of the 42 patients evaluable for efficacy, 34 (81%) demonstrated objective evidence of a response, with similar response rates being noted in patients with ovarian cancer and those with primary peritoneal cancer. At the time of publication, median progression-free survival had not been reached, but was greater than 16 months. Grade IV neutropenia was the most common toxicity (occurring in 64% of patients) and neuropathy was reported by only three patients (grade I=1; grade II=2). Hypersensitivity reactions were relatively common (34%) but did not result in the discontinuation of therapy.

### Phase III trial *vs* paclitaxel

The efficacy and toxicity profile of docetaxel–carboplatin has been directly compared with that of paclitaxel–carboplatin as first-line therapy for stage Ic–IV epithelial ovarian cancer in an international Phase III randomised trial conducted by the SGCTG. The trial, named SCOTROC (Scottish Randomised Trial in Ovarian Cancer), enrolled 1077 chemonaïve patients between October 1998 and May 2000 from 83 centres in 10 countries. Patients were treated with carboplatin to AUC 5 plus either docetaxel 75 mg m^−2^ infused over 1 h or paclitaxel 175 mg m^−2^ infused over 3 h. Survival and longer-term toxicity results were presented at ASCO 2002 (Vasey on behalf of the Scottish Gynaecological Cancer Trials Group, 2002). These results demonstrate that while the paclitaxel and docetaxel regimens are of similar efficacy, there are significant toxicity differences between the two therapies. The median reported follow-up in surviving patients was 21 months, with 94% followed up for more than 1 year. Docetaxel–carboplatin achieved similar median progression-free survival to paclitaxel–carboplatin (15.1 *vs* 15.4 months) and clinical response rates (66 *vs* 62%), but the duration of follow-up is currently insufficient to allow survival comparisons. Nevertheless, paclitaxel–carboplatin was associated with a significantly higher rate of grade II/III sensory neuropathy than docetaxel–carboplatin (30 *vs* 11%; *P*< 0.01), while docetaxel–carboplatin resulted in a significantly higher incidence of grade III/IV neutropenia (94 *vs* 82%; *P*<0.001) and febrile neutropenia (10 *vs* 2%; *P*<0.001), although these events were predictable and easily managed (Vasey on behalf of the Scottish Gynaecological Cancer Trials Group, 2002). Global quality of life parameters based on the EORTC QLQ-C30 instrument were comparable in both arms. However, using the ovarian-specific module OV-028 (Cull *et al*, 2001), patients reported significantly less severe symptoms of neurotoxicity (using a score based on tingling in hands or feet and numbness in fingers or toes) with docetaxel–carboplatin than with paclitaxel–carboplatin during treatment and also 6 months after randomisation (both *P*<0.001).

## SUMMARY

Over the last few years, the combination of a platinum compound such as cisplatin or carboplatin with paclitaxel has emerged as standard chemotherapy for advanced ovarian cancer (Kaye, 2000). Notwithstanding the clinical and survival benefits afforded by these new regimens compared with previous therapies, mortality from advanced ovarian cancer is high. Thus, research into new agents and new combinations continues apace with the objective of improving overall survival and reducing treatment-related toxicity. Docetaxel offers an alternative taxoid treatment to paclitaxel for use in this setting. Indeed, there is preclinical evidence that docetaxel has greater antitumour potency and a better therapeutic index than paclitaxel (Bissery *et al*, 1995), and its short 1-h infusion also offers a substantial clinical advantage over the 3- or 24-h infusion times required for paclitaxel. In clinical studies, docetaxel monotherapy has demonstrated good response rates and an acceptable toxicity profile in both paclitaxel- and platinum-refractory ovarian cancer patients (Kavanagh *et al*, 1996; Kaye *et al*, 1997; Verschraegen *et al*, 2000). Of particular note, neurotoxicity (a dominant side effect with both paclitaxel and cisplatin) is infrequent and mild with docetaxel, which implies that this drug is a promising new taxane for use in combination with cisplatin and other platinum compounds.

The incorporation of docetaxel into first-line platinum-containing regimens for advanced ovarian cancer has produced successful results. In Phase II studies, overall response rates of 69–74% were achieved with docetaxel 75 mg m^−2^ plus cisplatin 75 mg m^−2^; corresponding rates with docetaxel 75 mg m^−2^ and carboplatin to AUC 5–6 were 81–87%. The docetaxel–carboplatin combination proved to be better tolerated than the docetaxel–cisplatin combination (Vasey *et al*, 1999, 2001). A Phase III trial comparing docetaxel–carboplatin with paclitaxel–carboplatin suggests that the two taxane regimens are equally efficacious, but demonstrate clear toxicity differences (Vasey on behalf of the Scottish Gynaecological Cancer Trials Group, 2002). In particular, paclitaxel–carboplatin produced significantly more neurotoxicity, leading to early treatment discontinuation compared with docetaxel–carboplatin. While paclitaxel–carboplatin is currently the standard chemotherapy in the clinical setting, docetaxel–carboplatin is an impressive alternative. It appears that certain patient groups – for example, patients at high risk of developing treatment-related neurotoxicity – may benefit from receiving docetaxel as an alternative to paclitaxel in platinum-based regimens (Markman *et al*, 2001; Vasey on behalf of the Scottish Gynaecological Cancer Trials Group, 2002).
